# FlowCron - Increasing access to HPC by wrapping Globus into a function-as-a-service

**DOI:** 10.12688/wellcomeopenres.23491.1

**Published:** 2025-01-13

**Authors:** Dimitrios Bellos, James Allsopp, Elaine M. L. Ho, Tibor Auer, Gavin Yearwood, Andrew J. Morris, Mark Basham

**Affiliations:** 1 The Rosalind Franklin Institute, Rutherford Appleton Laboratory, Harwell Campus, Didcot, England, OX11 0FA, UK; 2 Advanced Research Computing, Computer Centre, University of Birmingham, Edgbaston, Birmingham, England, B15 2TT, UK; 3 University of Birmingham School of Metallurgy and Materials, Elms Road, Edgbaston, Birmingham, England, B15 2TT, UK

**Keywords:** High-Performance Computing, HPC, cryo-EM, Globus, Slurm

## Abstract

Despite significant investment in High-Performance Computing (HPC) clusters by funding councils, there are still many researchers whose workflows could not benefit from the computation speed that is provided by these clusters. Reducing barriers to entry for these researchers would accelerate their scientific throughput, since they will be able to respond to results in a timely fashion, improving either their protocols or correcting any problems that might have arisen. This improves the quality of science, and therefore the return on investment, in computationally-intensive areas such as Cryogenic Electron Microscopy (cryo-EM). This paper outlines a technique, FlowCron, for users to analyse their data on a HPC facility with minimal training, increasing accessibility. FlowCron transfers the responsibilities of installation and upkeep of data processing pipelines from users to HPC systems administrators, simplifies the set up of HPC pipelines, and makes pipelines as reliable as possible once set up. The work described here has software dependencies that are common to the majority of HPC clusters.

We achieve this by linking Globus and cron to produce an open-source system that requires little administrative support but provides a very easy way of running an analysis on a HPC system. The user starts the analysis through the Globus website and, when started, the data will be encrypted, uploaded to the HPC, analysed, and returned to the originating machine, along with a record of the analysis.

## Plain language summary

Despite significant investment by public funding councils in multi-million pound high-speed computing clusters, many researchers still do not utilise them in their work. By reducing any barriers to entry, the hope is that more researchers will increase their usage and productivity. This will accelerate their scientific throughput, allowing them to adapt to experimental results in a timely fashion, improving their protocols or correcting any problems that arise before significant costs are incurred. As a result, there will be a better return on the investment in those computer clusters and other equipment. This paper outlines a technique called FlowCron which allows users to analyse their data with minimal training. It allows the automatic transfer of data to these clusters, performs any computation to analyse and process the data, returns the processed data to the user, and then optionally deletes any data or files left in the cluster. Furthermore, all actions performed are documented and logged, encouraging reproducibility of the results and reducing effort associated with troubleshooting, maintenance, and support. Finally, FlowCron has ubiquitous software dependencies, making it simple to install and use.

## Introduction

Data has never been more available, with current instrumentation able to produce gigabytes of data and the algorithms used to process this data require huge amounts of computation, with applications ranging from Cryogenic Electron Microscopy (cryo-EM) to Large Language Models (LLM). This large-scale data and analysis is also moving beyond its traditional home in the science, technology, engineering, and mathematics (STEM) disciplines to encompass all research activities ^1^. A consequence of this is that there is a high demand for alternatives to the standard model of remotely accessing an HPC system using a UNIX-style terminal. Baskerville (EPSRC’s Tier 2 facility, run by the University of Birmingham) has already had success doing this by creating an OnDemand ^2^ portal so that users can use graphical user interface (GUI) apps they are already familiar with, such as Jupyter Notebooks ^3^, Fiji ^4^ and RELION ^5^.

Currently, UK funding councils invest significant amounts on HPC facilities, ranging from the Archer2 Tier 1 facility, through the nine Tier 2 facilities, (including Baskerville), to many smaller facilities within UK universities, such as the University of Birmingham’s BlueBEAR internal HPC system. In particular, the combined funding of all Tier 2 facilities was close to 43.8 million GBP. This is supplemented by research funds being spent on a per-job basis with Hyperscalers, such as Amazon Web Services (AWS) and Microsoft Azure.

To justify this increased investment in HPC and promote the utilisation of these facilities, we need to make it possible for users, who have good technical knowledge of their field but do not have prior HPC experience, to take advantage of these facilities with minimal help. Also, as academic HPC system administrator time becomes more and more indispensable, any system put in place that improves the HPC’s accessibility should be designed in a way that minimises the workload required in order to support it. This includes both the workload needed to set it up and to support it throughout its operational lifetime. Ideally, it should be a system that can be set up and administered by a trained HPC user, on behalf of other novice users possessing basic IT skills.

Hyperscalers have moved from simply offering virtual machines and popularised the concept of Function-as-a-Service (FaaS), with products like Amazon Lambda, where a stateless function, supplied by the user, is run on a remote computer on supplied data and the results are returned to that user. In this model, everything other than the function (e.g. hardware, operating system, libraries, and runtime) and the data transfers are managed by the hyperscaler. In this paper, it is this simplicity we are trying to replicate but in academic HPC facilities.

The task can be split into two parts:

- Transferring the data to and from the HPC facility quickly and securely.
- Triggering the execution of a series of commands on the receipt of the data, before triggering the return of the data to the user.

To perform the transfer, we use the University of Chicago’s Globus system ^6, 7^, which is an evolution of the GridFTP system ^8^. This provides not only very high sustained data rates between institutions but also federated security, a user-friendly interface, and a well-documented Software Development Kit (SDK). To provide an example regarding the data rate, during Globus’ usage between the research partners at the University of Birmingham and the Rosalind Franklin Institute, data rates that reached *∼*350MB/s over a week have been recorded. By using parallel transfers, it significantly outperforms traditional UNIX tools such as rsync and scp. It is also possible to configure the HPC Globus collection, so that all data transfers in and out of the HPC, are always performed using encryption. Finally, Globus apart from data transfers, also allows the user to perform basic access control actions e.g. creating new directories, deleting files, renaming files, etc.

Using Globus Flows, a developer can chain together a series of these access control actions to e.g. perform data transfers from one location to another whilst performing checks at each stage, set up recurrent transfers that auto-terminate if some conditions are met, etc.. These Globus Flows can then be published on an institutional level to authorised users of the same institution to use through the Globus website (however this feature requires that the institution has a Globus Subscription). Certain parameters, such as the destination path, can be left to be defined by the user when a Globus Flow is started, subject to checks defined in the flow. An advantage of Globus is that when running this flow the user doesn’t have to be connected to the system for the duration of the transfer, as with tools such as rsync. The user is periodically updated as to the progress of the transfer via email or through the web interface. Additionally, the use of encryption can be enforced by the owners of a Globus collection involved in a transfer, with no option for the user to circumvent this, allowing compliance with any regulatory requirements.

Although there is already another Globus system (Globus Compute, previously called FuncX) that integrates with Globus Flows and allows for running code on a remote system, we have opted against this due to the administrative costs of setting up the system and security issues with allowing Remote Code Execution (RCE). Instead, we use Globus to handle the initial transfer of the newly submitted job. The submitted job is in the form of a directory that has a specific internal directory structure, containing both a Slurm script and the relevant data files. Then after the transfer is complete, it is picked up by a cron job and processed using the HPC system’s Slurm scheduler. Then depending on the termination status of the job (successful or failed due to an error), the submitted job directory is placed either under a directory designated for successfully completed jobs or under a directory for designated for jobs that terminated due to an error. After this Globus transfers back any newly created or modified data files. This happens regardless of the termination status of the job allowing the users to read the error outputs in case of a termination due to an error. Finally, depending on the user’s choice, the submitted job directory and all its data can be deleted from the HPC to free up storage or they can be left on the HPC so that the job’s output files can used as inputs to subsequent jobs.

Although this has been designed and tested for use on the Baskerville HPC, we hope that it will be remarked as useful by other members of the HPC community, so it finds adoption and be implemented on other HPC clusters as well. The only requirements should be:

1. BASH (>4.0). Bash scripts can be run on the HPC.
2. Slurm ^9^. Job submissions on the HPC are done via a Slurm job scheduler.
3. cron. The cron job scheduler is available on the HPCs login nodes.
4. Globus. The HPC should have a Globus collection so users can upload data to the HPC. Also, the users can install Globus on their local workstations. Typically HPCs install Globus as a Globus Connect Server (GCS) and they can even set it up with an OpenID Connect (OIDC) layer so their access policies are followed even when users access the HPC via Globus. Moreover, HPC’s can setup a single GCS collection without requiring a Globus Subscription, however if the same institution (HPC or the user’s institution) requires more than one Globus collection this requires a Globus Subscription. Users may have the ability to upload/download their data from their workstations via an institutional GCS collection or by installing Globus Connect Personal. Server Deployment of Globus requires significant changes to firewall rules to be set as explained here ( GCS firewall rules, GCP firewall rules) in order to operate normally.

An optional requirement is a Globus Subscription from the institution that wants to use FlowCron. Although everything here can be run without having a Globus Subscription, it offers the following advantages:

1. The users can create Globus groups. This is particularly useful for the members of the institution who are Principal Investigators (PIs) or managers on one or multiple projects set on the HPC. This way they can create a Globus group per project and add in all the members of the project.
2. The users can have more than one Globus Flow. This allows PIs or managers of HPC projects to create a FlowCron Globus Flow per HPC project.
3. The users, for their Globus Flows can give a "Starter" role to a whole Globus group, enabling the distribution of a launchable Flow to everyone in the group. These PIs or managers of HPC projects can share their FlowCron Globus Flows with the appropriate Globus group.
4. The users who created Globus groups, can add and remove members. These PIs or managers of HPC projects can then control who can use a particular FlowCron Globus Flow.

## Methods

### Implementation

The implementation consists of two parts: the Globus Flow JSON files, which are written using the syntax of Amazon State Language, and a series of bash scripts to be installed on a server. These can be found at "FlowCron-Globus_Flow-side" ^10^ and "FlowCron-HPC-side" ^11^ GitHub repositories respectively.

There are two Globus Flow JSON template files:

- An input schema that defines the flow’s inputs and their types, and performs validation. This is then used to construct the flow’s user interface.
- The definition file. This JSON file contains a series of states, each of which is an action. Each state defines whether it is a start or end state, or to which states this state passes control to on completion.

These are used by Globus to create the flow.

State types are:

- Action (not in Amazon State Language)
- ExpressionEval (not in Amazon State Language)
- Pass
- Wait
- Choice
- Fail

The choice state allows for logic within the flow itself. It’s important to note that actions available to the Action state are quite limited compared to what you can do from a terminal. Some of the available actions are transfer, delete, make directory, manage permissions and get file status.

These templates are used by the script **construct_new_instance_FlowCron_configs.sh**, which asks for user input to produce concrete schema and definitions from the templates.

On the server side, a cron job launches a Bash script ( **CodeToRun/cron.target.sh**) recursively at a user-defined time interval, following a successful run of the **setup.sh** script. Bash was chosen for this due to its ubiquity on HPC machines without having to load any modules or other code to run it, as would be the case with Python for example.

Each job to be analysed requires a directory structure to be created for it, called a **Unit of Work(UoW)**. This consists of a directory containing the directories **scripts** and **data**. It is this parent directory that is transferred to the HPC facility using Globus. The user places their data in the **data** directory and a Slurm script in the scripts directory. The maximum size of a Unit of Work is dependent on the project’s storage quota on the HPC machine, which is currently 1TB for Baskerville. The code searches the **scripts** directory for a single file matching the following conditions;

- Starts with **#!/bin/bash**.
- Has at one or more **#SBATCH** directives.

If these conditions are not met the Unit of Work is marked as failed.

The **Unit of Work** is uploaded by the flow to a directory called **UploadedFiles**, created in the location where **./setup.sh** was run. The **CodeToRun/cron.target.sh** script looks for Units of Work in this directory, and when found copies them to **CodeToRun/slurm** and submits the script found in **scripts/** to the Slurm job queue. In doing this it starts a second job that runs the **cleanup.sh** script as a job dependency of the first job. The dependency structure guarantees that the **cleanup.sh** script will be run regardless of the exit state of the user’s job.

If the first job finishes with an exit code of **0:0** in the Slurm output file, indicating success, the second job ( **cleanup.sh**) copies the Unit of Work and all files created as part of the process to **AnalysedFiles**. Here it awaits download back to the user’s computer by Globus. Should another exitcode or none be found, the Unit of Work is transferred to **FailedJobs** where it will remain on Baskerville for triaging. To ensure that the project’s storage quota will not be filled retention and soft-delete policies can be put in place. By soft-delete, we mean that instead of immediately deleting the Unit of work, we move it to a separate "Recycling Bin" directory after a certain time period has passed, and only then finally delete the Unit of Work after another period of time has passed.

An individual Unit of Work contains the output from Slurm, but more extensive logs relating to the running of the instance of FlowCron can be found in the **Logs** directory. All of the transfers, and the actual running of the job, are protected by sentinel files and directories to prevent the system from moving a Unit of Work to the next step prematurely. For instance, **CodeToRun/cron.target.sh** refuses to begin analysis of a job until the **sentinels** directory is created in the Unit of Work, a task which is done by the Globus Flow action occurring directly after a successful upload. Similarly, a file is then copied into that sentinels directory once a copy to the **CodeToRun/slurm** begins and is deleted once that is finished. Similar mechanisms exist whilst the Unit of Work is being run by Slurm and when it is being copied from the work area to either **AnalysedFiles** or **FailedJobs**.

**Figure 1.**
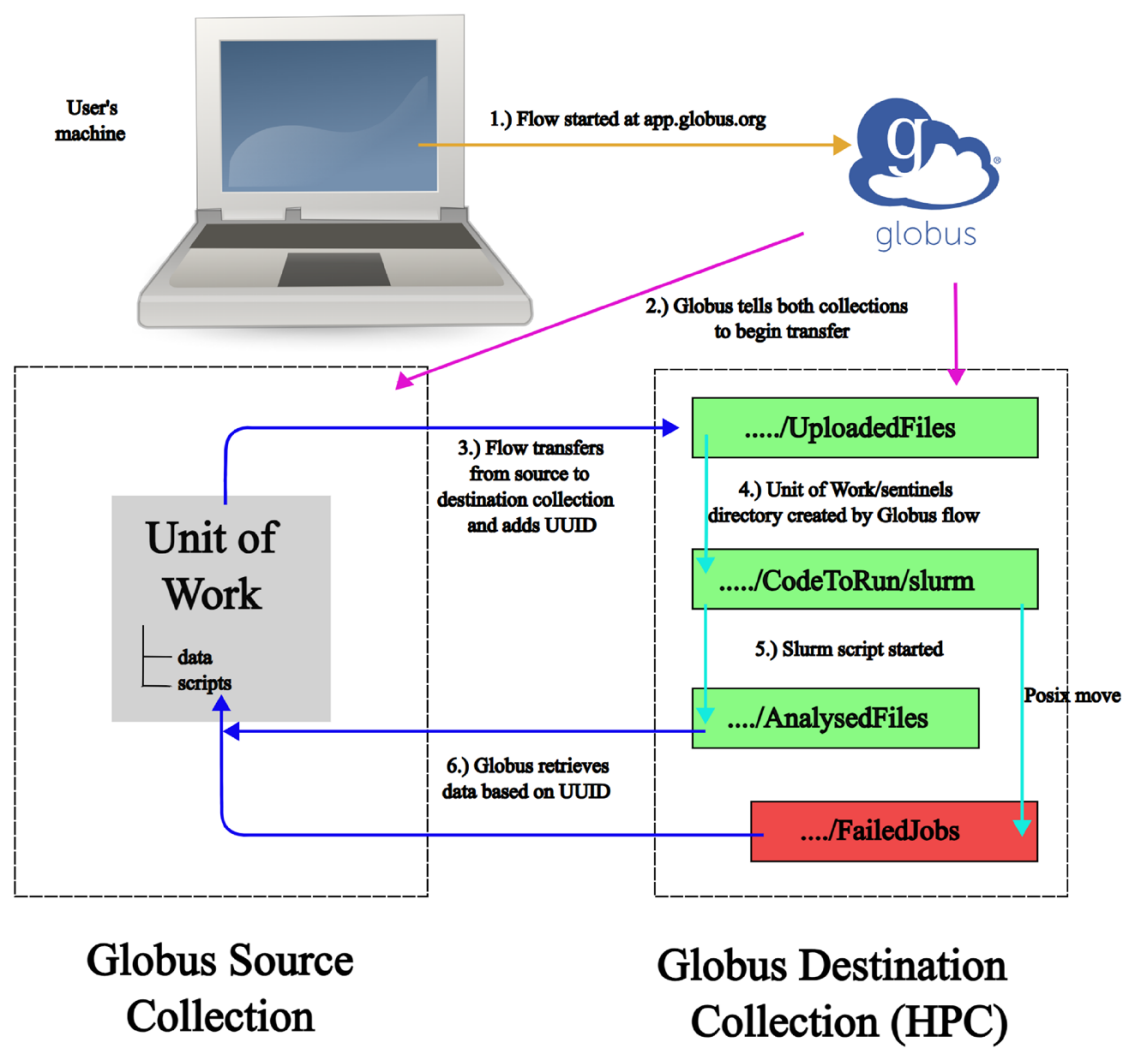
Showing the FlowCron process across the four systems. The gold arrow shows a user logging into app.globus.org and starting the flow. The magenta arrows show Globus setting up the communication between Globus Collections. Blue arrows show a Globus transfer between Collections and a cyan arrow represents POSIX moves, which move a Unit of Work between directories.

The full process can be seen (without error branches) in Figure 1.

With the use of sentinels, one could chain FlowCron instances together for more complicated analyses, with one FlowCron’s AnalysedFiles being the next FlowCron’s UploadedFiles. This might be necessary when running jobs that require different Slurm resources at different stages.

### Operation

The system is designed to be as quick to set up and use as possible, with any errors being transparently handled. Detailed logs are kept for a default of one week allowing issues to be quickly diagnosed and fixed. Error handling is designed to retain evidence of any problems to aid with debugging, such as with Slurm scripts and intermediate files.

Setting up the system follows these steps:

1. A HPC project PI or manager should install the HPC-side code ^11^, thus setting up the cron service on the HPC. This is done by checking out the repository and running the **setup.sh** script, defining the path where the installation occurred (which should be in a HPC project directory) and the name of this FlowCron installation (a name to easily identify that FlowCron installation is regarding a particular HPC project).
2. A HPC project PI or manager should run the Globus Flow side code ^10^, to generate the JSON files of FlowCron’s Globus flow. This is done by checking out the repository and running the **construct_new_instance_FlowCron_configs.sh** script. Any info provided with the script prompts should agree with the information provided in step 1. If there is not an institutional Globus Subscription, the HPC project PI or manager should distribute the generated JSON files to all users who need to use FlowCron.
3. (Optional. There should be an institutional Globus Subscription) Create a group on the Globus website and add any users you wish to run the flow to this group. The users will also have to be members of the project on the HPC cluster. This is so that FlowCron’s Globus Flow can access the cron service that is installed on the project directory on the HPC.
4. Create FlowCron’s Globus Flow on the Globus website, using the information from steps 1. and 2. If step 3. has been performed, then users do not have to create FlowCron’s Globus Flow (aka the HPC project PI or manager did not distribute the JSON files). In this case, the HPC project PI or manager can assign the Globus group they created in step 3. with a "Starter" role on FlowCron’s Globus Flow. By managing the members of the Globus group they can manage who can use FlowCron. If step 3. was not performed, then every user has to create FlowCron’s Globus Flow using the JSON files provided by the HPC project PI or manager.

Using the system requires creating a Unit of Work, and visiting the Globus website to start the flow, giving the location of the Unit of Work on the source collection.

### Setting up HPC-side

The code for the HPC part of the system is stored on a publicly accessible GitHub repository ^11^. Once checked out to the HPC system, a user runs **./setup.sh**, which takes the user through configuring cron and creating all of the relevant directories.

When the **setup.sh** is run, the user is asked:

- What should this instance of FlowCron be called?
- What Slurm account would you like this to run as?
- What Slurm QoS would you like this to run as?
- How often should this cron job run? (In other words, how often the cron service checks if new Units of Work have been uploaded).
- How many days should pass before we soft-delete completed or failed jobs?
- How many days should pass (after copying to soft-delete) before we finally delete completed or failed jobs?
- Should a timestamp be added to Uploaded directories?

By soft-delete we mean temporarily moving the Unit of Work directories to a "Recycling bin" directory before eventual deletion. The term delete or hard-delete signifies the permanent deletion of the Unit of Work directories.

The name of the FlowCron instance is important as this means more than one instance (typically installed on different project directories on the HPC) can be used by the same user (e.g. if the user is a member on multiple HPC projects), without conflicts. The supplied Slurm account and QoS (that are typically HPC project specific) are substituted into the **cleanup.sh** script. As HPC facilities have strict limits on storage space, there are soft-delete and hard-delete time limits, these should be set to zero during setup if the user wants to disable them. Adding a timestamp causes the supplied Globus Flow to fail, so we strongly suggest that this is set to no. In other situations, however, such as testing or in a custom setup, the user might want to set this to yes so that a timestamp is added to each uploaded Unit of Work. This prevents the directories from two Units of Work with the same name getting their files combined when a Unit of Work with the same name is uploaded before the existing Unit of Work has been moved for analysis. The Globus flows code adds a UUID to the directory name on upload ensuring uniqueness.

It is strongly recommended, on a system with user and project directories, to run this **setup.sh** in a project directory rather than a user directory to avoid hitting a user’s quota limits. This also allows other users to read logs, etc.

For reference, the settings from **setup.sh** are saved in a user’s $HOME/.config/flowcron directory. To change these values you can rerun setup.sh. For finer-grained control a lot of these variables are stored in **environment_variables.sh**.

For testing purposes, the HPC-side repository contains an example **Unit of Work**, called **example_unit_of_work** containing an example Slurm script **edit_this_slurm_script.sh**, which outputs some information about the working directory. Before running, this requires a user to edit the Slurm file, putting in valid QoS and account values. This script can also call other non-Slurm scripts and there are no limitations to either single-node or single-GPU jobs.

The only other constraints on the Slurm script, over and above those imposed by Slurm or the HPC facility, are the following:

- Do not use environment variables such as **$USER** and **$HOME**, they are unlikely to be valid as the script runs as the user who ran setup.sh. We try to detect these and fail the script to save resources.
- Do not try and customise Slurm output (e.g. #SBATCH –error <filename>), this will be overwritten.
- Use relative paths to refer to files within the **Unit of Work**.
- Use absolute paths to refer to files elsewhere on the HPC cluster, such as large data sets shared between jobs, such as a protein database. You can find this by changing to the resource directory and running **pwd -P**.
- Avoid using the cd command as this might have unintended consequences. You could, in the beginning of the command section of the script, create symlink directories in the **Unit of Work**, and then use relative paths in the subsequent commands.

The last two items are required as the Unit of Work is moved between directories during analysis and a user cannot assume any locations relative to one another.

### Setting up the globus flows side

The code to generate custom Globus Flow JSON files for FlowCron, is stored on a publicly-accessible GitHub repository ^10^. To run it requires information from the HPC setup and information about the UUID HPC’s Globus Collection before starting, so make sure you’ve done that first.

Furthermore, to run the code the user would require a machine that can run Bash shell script (e.g. on a Ubuntu machine, or a Windows PC with Windows Subsystem for Linux installed, etc.) and you can open a browser on.

Once the code is downloaded locally and the user navigates within the code directory, they may run the **construct_new_instance_FlowCron_configs.sh ** script.

**Figure 2.**
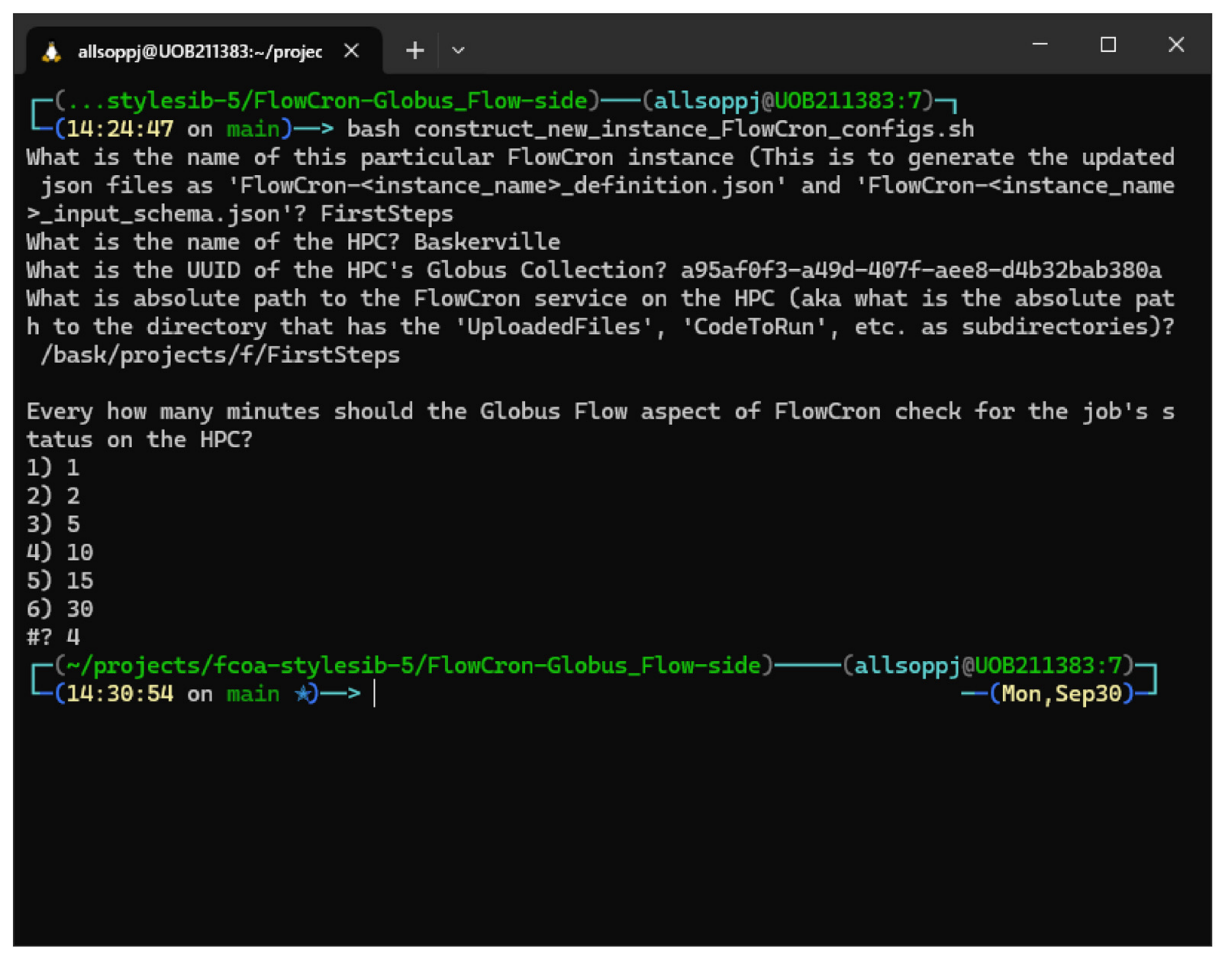
Show the input given to the construct_new_instance\FlowCron\configs.sh script from a bash command line.

The different prompts that will asked are shown in Figure 2.

The user will be asked for a name for the custom Globus Flow they are about to generate, typically they should use the same name they used when setting up the HPC-side. This is not mandatory but helps tie things together (Question 1). Similarly, the name of the HPC (in our case it was "Baskerville") is again an identifier for the users. This means that all FlowCron’s Globus Flow logs and transfer labels will use the argument provided to refer to the HPC (Question 2). The HPC is definitively identified by its Globus Collection UUID (Question 3). The absolute path is the path to the directory where the HPC-side cron service was installed. It should point to the directory that contains the **UploadedFiles** directory, **CodeToRun** directory, etc. (Question 4). If you log in to the HPC and navigate via the terminal to this directory, the command **pwd -P** will give you the answer resolving any symbolic links. Setting the value for the time to check the job’s status (Question 5) depends on the length of time you expect the job to take to run. For a short job, you want this period to be relatively short so that it’s not just waiting there, conversely for a longer job, checking regularly is a waste of resources.

**Figure 3.**
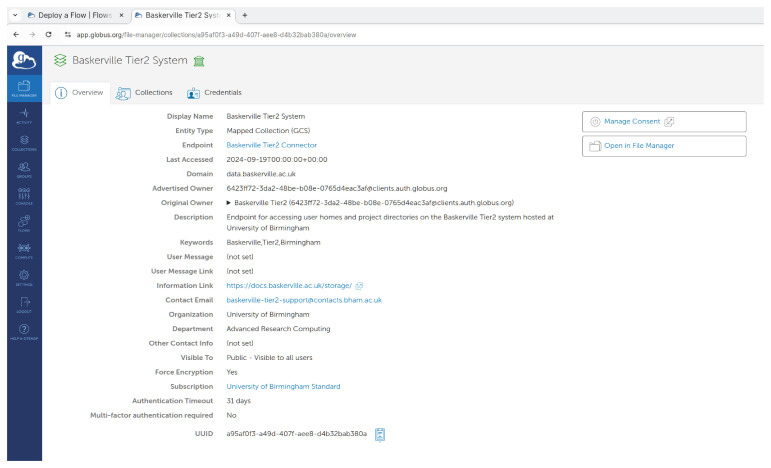
Shows the app.globus.org page showing the Baskerville collection’s UUID.

The Globus UUID can be found from app.globus.org, by clicking on **"Collections"** in the left side menu and then going to collection details on the right. The output from this is shown in Figure 3.

After running the **setup.sh** script, a directory will have been created next to the **setup.sh** file with the name you gave as the answer to Question 1. Inside that directory will be two files,

- **FlowCron-<Name given>_definition.json**
- **FlowCron-<Name given>_input_schema.json**

Once the JSON files have been generated, everything is ready in order to create the flow using these files. This can be done by visiting the Globus website and creating the flow using the Globus website UI.

**Figure 4.**
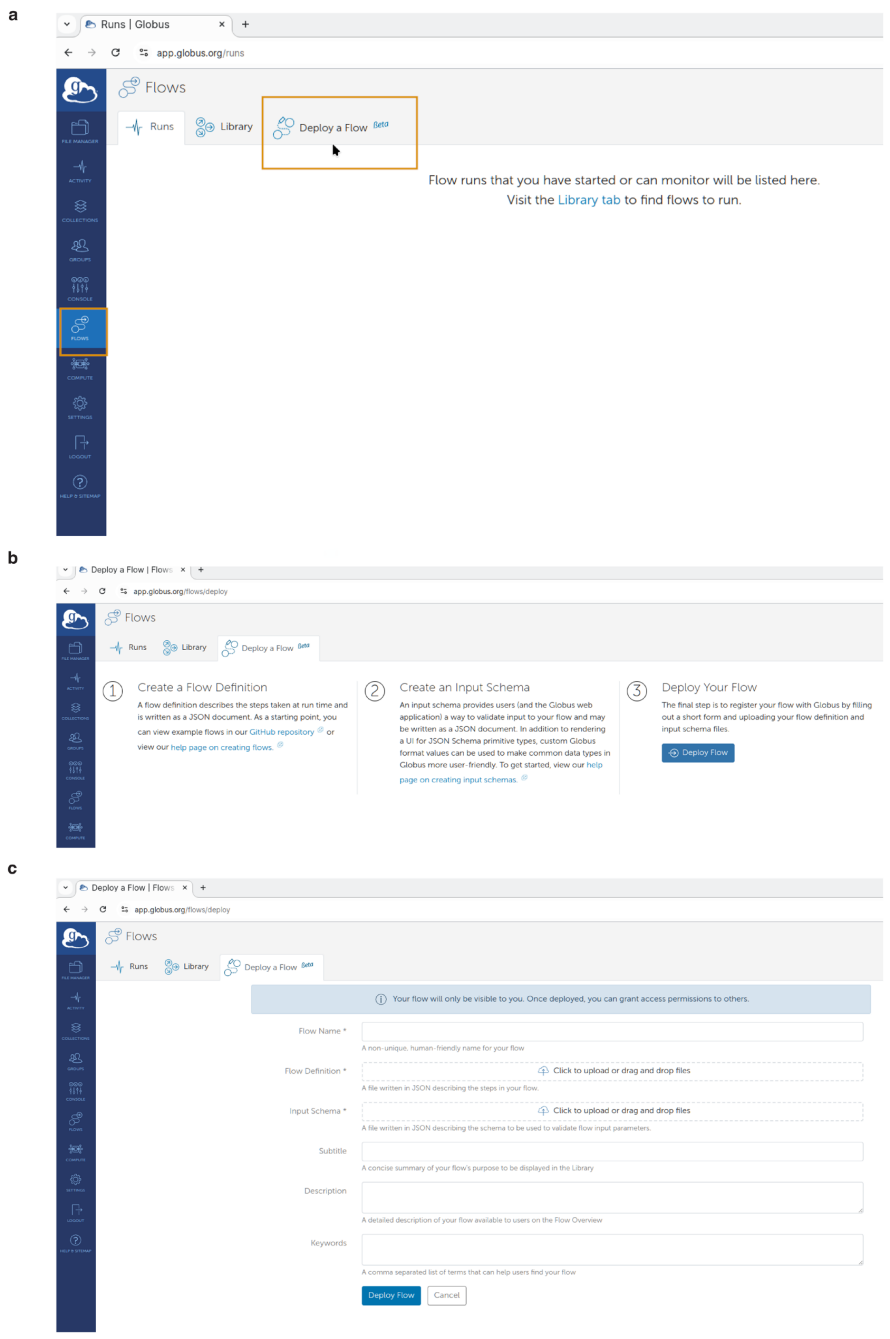
Creation of a Globus Flow for FlowCron on the app.globus.org website by uploading the FlowCron generated JSON files. ( **a**) Step 1: Creating a flow on app.globus.org, ( **b**) Step 2: Deploying a flow on app.globus.org, ( **c**) Step 3: Configuring a flow on app.globus.org.

To perform this as shown in Figure 4a on the left side menu, the user may select **"Flows"** and then click on **"Deploy a Flow"** tab.

Then as shown in Figure 4b they should click the **"Deploy Flow"** button.

Finally, in the UI shown in Figure 4c they should use the JSON files by uploading them on the Globus website. They should upload or drag-&-drop the **FlowCron-<Name given>_definition.json** on the **"Flow Definition"** field and they should upload or drag-&-drop the **FlowCron-<Name given>_input_schema.json** on the **"Input Schema"** field. For the flow name, they may use the same name used when they run the setup.sh. The user may additionally enter any appropriate values to the other boxes so that they can easily find the flow in the future. Create the flow by clicking the **"Deploy Flow"** button.

An alternative way to create the flow is by installing Globus CLI and then us the Create Flow Globus CLI command, but this requires a user to have some UNIX and command line knowledge.

If the user’s institution does not own a Globus Subscription, every user will have to create their own flow. For speed and reliability, the JSON files may be generated once and then shared amongst FlowCron users. Without a Globus Subscription, each user can only create a single flow. This presents difficulties if the user participates in multiple projects in the HPC as a different FlowCron will have to used per project. In this scenario, they will only be able to use the flow for only a single FlowCron installation at a time.

It is expected that the Principal Investigator (PI) or manager of project in the HPC will setup FlowCron both from the HPC-side and also create and distribute FlowCron’s Globus Flow.

**Figure 5.**
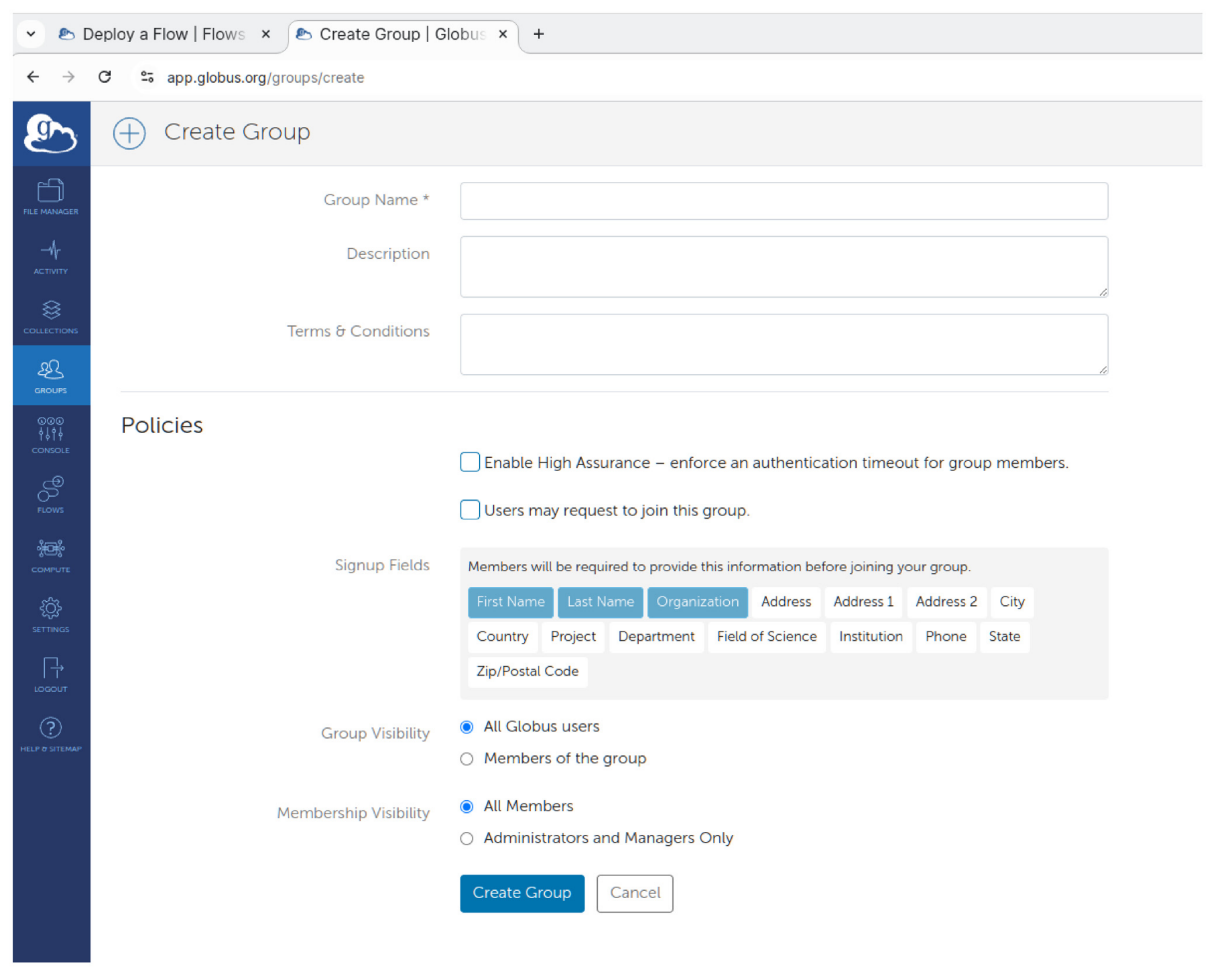
Creating a group to use the flow on app.globus.org.

Once they create the flow with the process described they earlier, they should continue with creating a Globus group, and invite all the users that are meant to use FlowCron’s flow. To perform this as shown in Figure 5, once they login to the Globus Website, on the left side menu they should select **"Groups"** and on the next page, click on the **"Create new group"** button. Once they fill all relative fields they can click **"Create Group"**. Here they can learn more about how they can Manage a Globus group.

**Figure 6.**
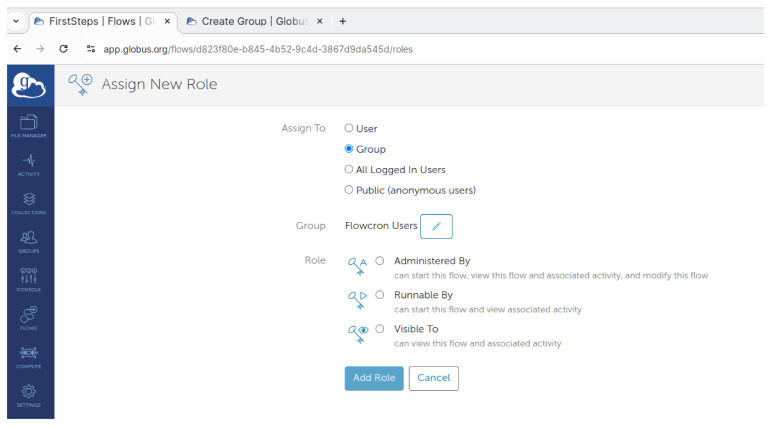
Adding a group to the flow on app.globus.org.

To publish a FlowCron flow to the group, on Globus Website select **"Flows"** on the left side menu and click on the **"Library"** tab. They can narrow down the resultant list of flows using **Administered by me**. Select the FlowCron flow to be published, click on the name of the flow, and then click on the **"Roles"** tab. As shown in Figure 6, select **"Group"** in the **"Assign To"** section, and then press the **"Select a Group"** button to select the group to which they want to publish the FlowCron flow. They should also select the **"Runnable by"** option for all members of the group to be able to run the flow, and then click the **"Assign New Role"** button. Once all is set they should click the **"Add Role"** button. Once this is done the flow is published to the group and they can now use it.

Administrators of the Globus group will also be able to remove and add members, therefore controlling who can use the FlowCron flow.

### Using FlowCron as user

**Figure 7.**
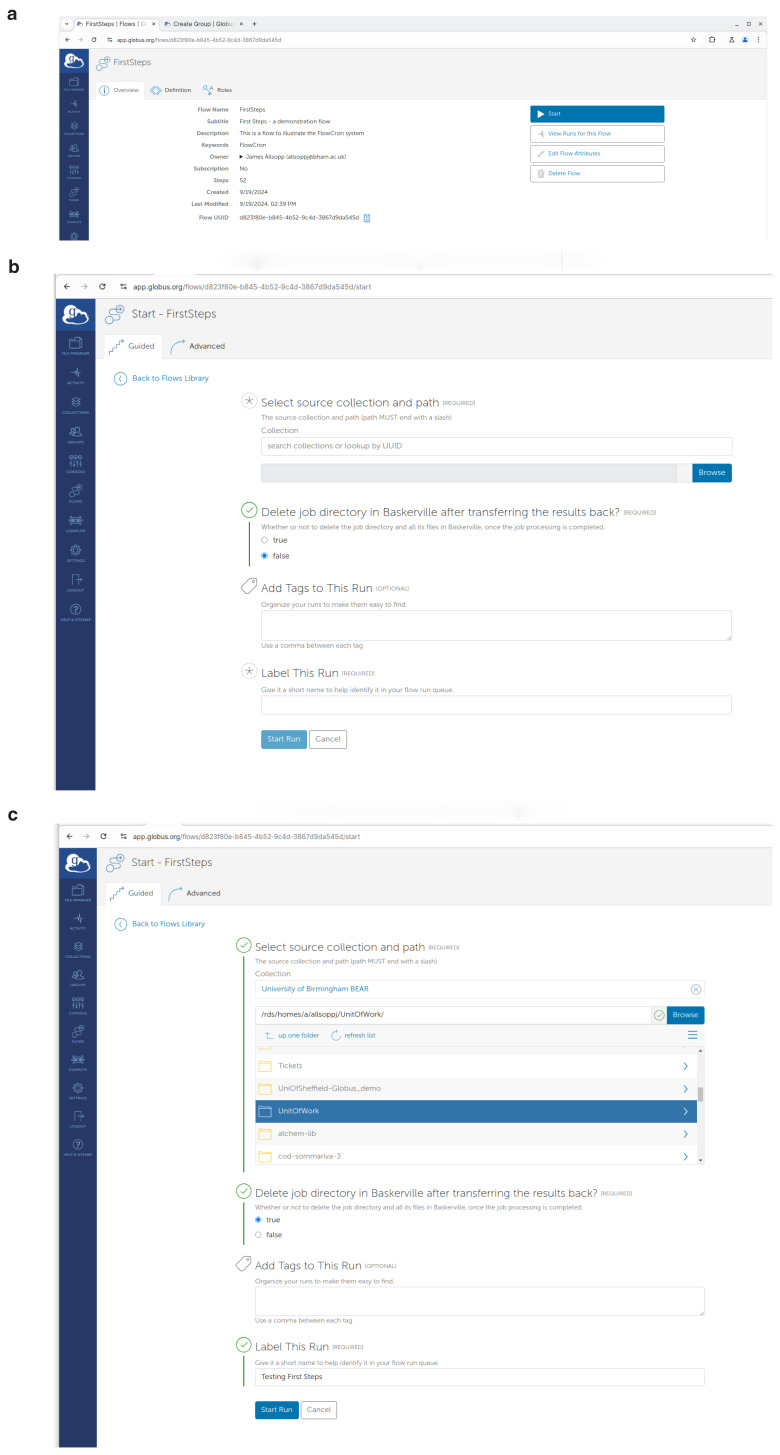
Using the FlowCron Globus Flow on app.globus.org to start a new "run" of the flow and submit a Unit of Work to be analysed on the HPC. ( **a**) Starting a flow called First steps app.globus.org, ( **b**) Form to start the flow app.globus.org, ( **c**) Filled in form, showing how to start the flow. app.globus.org.

To submit a Unit of Work to be processed, a user should visit app.globus.org, login, select **"Flows"** from the left side menu and find the FlowCron flow in the list. You can either use the search box, or narrow down the resultant list using the **"Administered by me"** (if you set the flow up), or **"Runnable by Me"** checkboxes. Once located, by pressing the **"Start"** button on the right, they will see the screen shown in Figure 7b. Alternatively, by clicking the title of the flow they can find more information as shown in 7a, and start the flow by clicking the **"Start"** button on the right.

Figure 7c shows where you enter the information with which to run the flow. For the collection you can either use the UUID of your source collection, or type the name, and the user can click browse to find the Unit of Work in the collection. If the Unit of Work is on the users’s local machine, use **Globus Connect Personal** (GCP) to set up a Globus collection on their personal machine, and search for the collection based on the name chosen when setting up GCP, which is usually the machine name. They will also need to decide if they want the processed Unit of Work to remain on the HPC. It can either remain on the HPC for further analysis, or be deleted to free up storage quota. A title label should be given to this "run" of the flow so it can be easily found in the logs. Finally, they should click the **"Start Run"** button to submit the Unit of Work.

**Figure 8.**
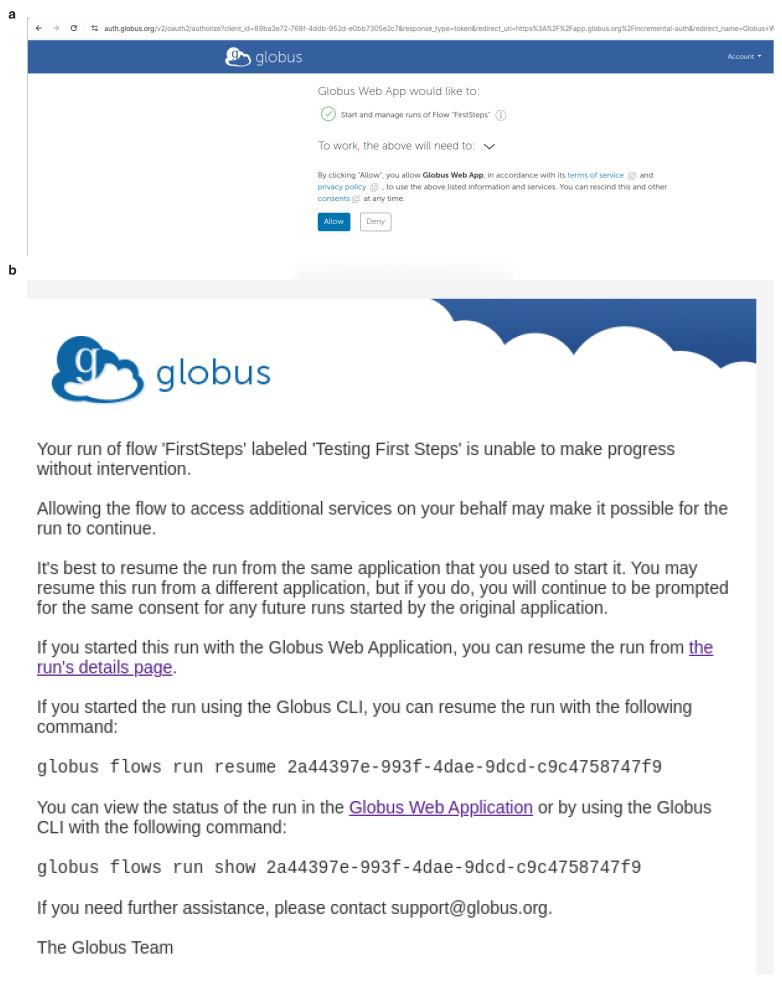
When running a Globus Flow for the first time it requires the user to consent to use it. Moreover, when the authentication session to access either the source or destination Globus collection expires, you will be asked to consent and/or provide credentials to access these collections again and thus allowing also the flow to access them. If you are not logged in to Globus, warning emails will be sent to the email address of the user’s Globus account. ( **a**) Authorise consents app.globus.org, ( **b**) Reviewing consents email app.globus.org.

After a flow "run" has started, you may be asked on the website to supply consents to allow the "run" to proceed. An example is shown in Figure 8a and you should click **Allow**. You will be asked to authorise these consents the first time you use a FlowCron Globus Flow. Then periodically when the authentication session to access either the source or destination Globus collection expires, you will be asked to consent and/or provide credentials to access these Globus collections again, thus allowing also the flow to access them. In the case of FlowCron, the source collection is the collection used by the user to upload a Unit of Work and the destination collection is the Globus collection of the HPC. Should you not be logged into the Globus website, you will receive an email as shown in Figure 8b.

**Figure 9.**
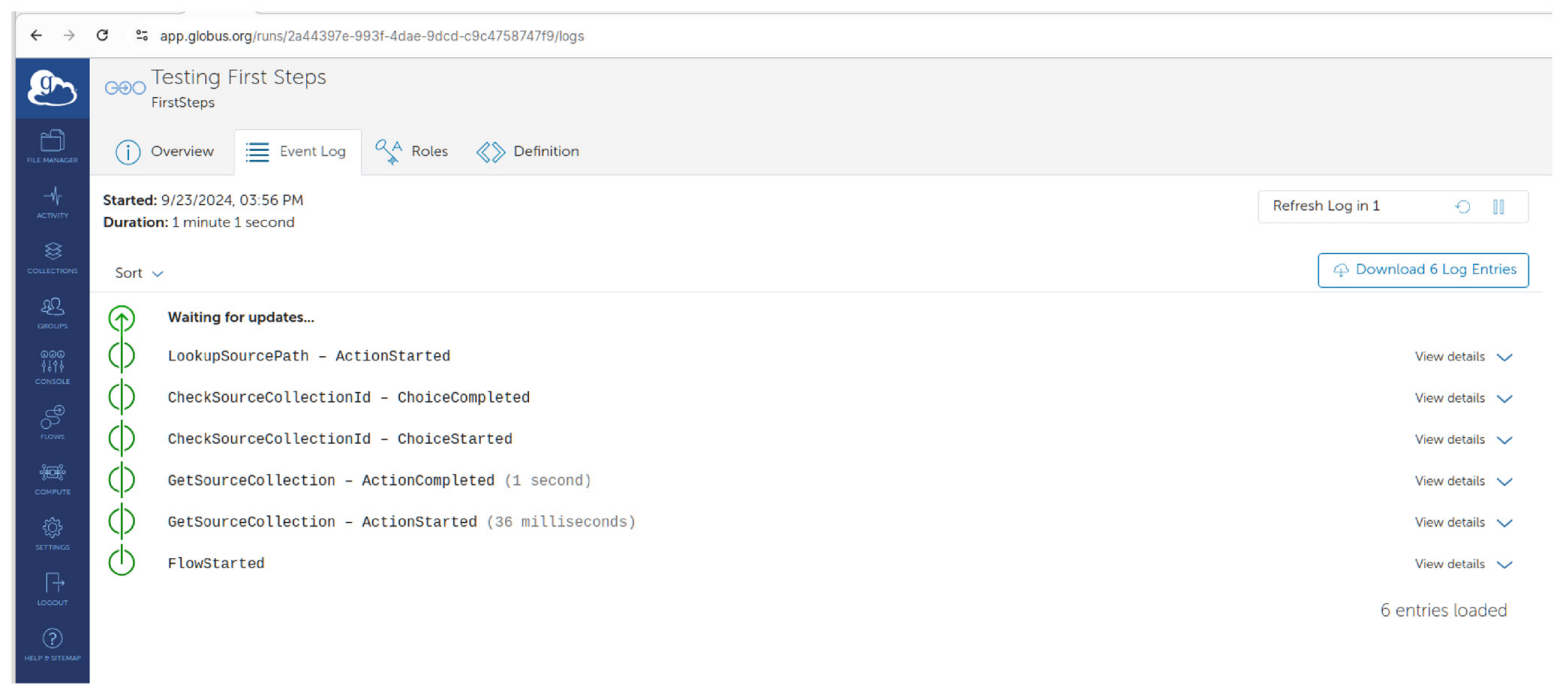
In progress log - app.globus.org.

You can track the progress of the flow’s run on the logs page as shown in Figure 9.

When the job described in the Slurm script which is included in the Unit of Work, terminates in the HPC, the processed data and Slurm output files of the Unit of Work should now be transferred to the user’s source Globus collection and path so they can view them. If the job terminated with an error code, the user should inspect the generated Slurm output files to diagnose the issue. They may also need to check the **FailedJobs** directory and the **Logs** directory in the cron service installed on the HPC.

## Use cases

The Rosalind Franklin Institute is a world leader in structural biology and makes extensive use of Cryogenic Electron Tomography (cryo-ET) ^12^, which is a form of cryo-EM for collecting 3-D images of biological samples at high resolution. Cryo-ET produces data files with sizes of the order of 500GB to 1TB for a single sample, and analysing this data requires several steps of varying complexity and computational intensity. Most of the steps can be accelerated with the use of a single or multiple GPUs, some require machine learning, and a few of them require processing on CPU cores. Because of this, it can be highly beneficial to analyze cryo-ET data in HPCs. However, most biologists who are sufficiently skilled to the software for the analysis of cryo-ET data (e.g. RELION ^5^), know how to do so only on single machines with a desktop environment, and they are not familiar with using HPCs via traditional UNIX and Slurm commands. Therefore, operating software like this via FlowCron is a prime candidate to test it.

We have tested FlowCron on two different mainstream use cases, performed predominantly using the RELION ^5^ software package.

### Preprocessing of cryo-EM data

Processing cryo-ET processes starts with the same pre-processing steps to obtain tomograms - motion correction, contrast transfer function (CTF) correction, alignment of the tilt series, and the reconstruction of tomograms from the aligned tilt series. The pipeline in this use case preprocesses cryo-ET data using a range of software programs detailed below:

1. Raw cryo-EM micrographs imported into RELION ^5^.
2. Motion correction of micrographs using the RelionCorr algorithm (a RELION CPU-based implementation of the UCSF Motion-Corr2 ^13^ algorithm).
3. Estimation and correction of the Contrast Transfer Function (CTF) using the CTFFind4 ^14^ algorithm.
4. Tilt series alignment and tomogram reconstruction using the AreTomo ^15^ software package.

This multi-step process can take several days to complete, and researchers have to manually run each step in sequence. In particular, the steps to run this pre-processing pipeline manually are:

1. Transfer the raw cryo-ET data to Baskerville HPC using Globus.
2. Wait until the data transfer is complete.
3. Once the Globus transfer is complete, processing steps are set up either using the RELION interactive app or using a SLURM script that needs to be prepared and submitted.
  (a) Users choosing to construct and submit a Slurm script need to have been trained to use Slurm commands and edit text via a Linux terminal.
  (b) User opting to use RELION as an interactive app will perform all preprocessing steps as separate Slurm jobs, so there might be a delay between running subsequent steps.
4. Once all pre-processing steps are complete, it is necessary to transfer of the tomograms out of Baskerville using Globus to visually inspect the datasets.

In our tests, we preprocessed the EMPIAR-11963 dataset (see Source Data), by allocating overall 3 GPUs (each being a Nvidia A100, VRAM 40GB) and 108 CPU cores on Baskerville. The total time required to transfer all the raw data to Baskerville, preprocess them, and then transfer them back was 19 hours and 15 minutes, which is significantly less than the multiple days required if done manually.

It is important to mention here that the goal of FlowCron is to reduce the time required to process data on an HPC, by automating the manual steps of transferring data to the HPC, job submission, transferring processed data back from the HPC, and cleaning up any leftover data that are not needed on the HPC. The delay between the different steps is determined solely by the readiness of the user. This makes it very difficult to pinpoint exactly how much faster operations are completed via FlowCron vs manually.

Apart from speed improvements, the use FlowCron offers additional benefits. In particular, due to verbose logging of the various actions performed, and the ability to store in the HPC all submitted Units of Work, it is much easier to reproduce results if the needs arise. This can be helpful both in situations when an issue has to be diagnosed or when the same processing has to be repeated on the same or new data. Futhermore due to the comprehensive logs collected it is easier for HPC administrators to maintain and support FlowCron.

### 3D classification of cryo-EM data

One of the most popular steps of analysing cryo-EM data with RELION is to perform 3D classification of the detected particles. To determine how user-friendly FlowCron is, it was tested by a researcher from the Rosalind Franklin Institute who had no prior knowledge on how to use FlowCron. By following the instructions in the README.md file of the FlowCron’s Globus Flow repository ^10^, the Franklin user, Gabryel Mason-Williams ^16^ (see acknowledgements), was able to transfer all his data (they used the same data as in 17) to Baskerville, perform 3D classification with RELION and transfer them back. According to him:

- *"I used FlowCron to run RELION benchmarking tests. It was very intuitive and helped reduce the barrier to entry for using HPC."*
- *"Overall this is a great project that should help improve the uptake of HPC usage, as it removes the need for knowledge of SSH and the command line, often a daunting experience for people with little computer experience."*

One of his criticisms was the need to know how to create a Slurm script. However, this can easily be resolved by the creation of a library of template scripts for the most commonly used processing operations. Then a user will only need to edit a template script each time to ensure that the correct input filenames are used. This can be avoided if the user uses a standard input configuration. Furthermore, the generation of a new template will be infrequent enough to justify asking for help from an experienced Slurm user. Because of this FlowCron reduces the support required by the user, as it is simpler, quicker, and requires fewer adjustments to FlowCron rather than doing everything from scratch for every analysis.

Overall, for the processing 2 GPUs (each being a Nvidia A100, VRAM 40GB) and 72 CPU cores were allocated overall on Baskerville. The total time required to transfer all the raw data to Baskerville, preprocess them, and then transfer them back was 56 minutes. For this benchmarking only a small dataset of 50GB was used and running only for 5 iterations (typical 25 iterations are used in RELION 3D classification). This explains why it was completed in under an hour. For typically sized cryo-EM jobs, with larger datasets and a higher number of iterations, this processing requires multiple hours or even a day. This operation may not have a lot of delays if done manually; however, FlowCron allows users to maintain focus and provides a smoother experience compared to the user manually performing the processing steps in succession.

## Discussion

Based on the real-world examples from one of the UK’s foremost cryo-EM research centres we show our work can make a significant contribution to improving access to HPC resources while improving the efficiency, robustness, and user experience of a common typically manual process. In addition to streamlining users’ workflows, there is a considerable time-saving, which allows users to be more responsive, improve their methodology in the light of prompt results, and allows more effective use of expensive facilities, such as electron microscopes. It is important to note that this software can be applied to any field, with computationally intensive tasks that need to be carried out repeatedly. An additional benefit is the clear audit trail making the data products more transparent and thus more reproducible.

Since the software tool presented here offers a novel feature: the ability to access an academic HPC with a function-as-a-service, there was not the need to mitigate any bias or unwanted sources of variability in the results. Lastly, open source datasets were used for this work (see Source Data).

## Ethics and consent

Ethical approval and consent were not required.

## Data Availability

In the following section, we cite the raw data used for our experiments/use cases. All raw data are already published and below we provide citations for all of them.

## Source data

The raw cryo-EM datasets used in this study have been deposited to the Electron Microscopy Public Image Archive (EMPIAR) ^18^, the Electron Microscopy Data Bank (EMDB) ^19^, and the Protein Data Bank (PDB) ^20^:

- Cryo-electron tomography on plasma FIB lamellae of HeLa cells ( https://doi.org/10.6019/EMPIAR-11306) as described in the Berger *et al.* ^21^.
- Also, the following 4 datasets described in the Wong *et al.* ^17^ were used:
  – Cryo-EM structure of the Plasmodium falciparum 80S ribosome bound to the anti-protozoan drug emetine ( https://doi.org/10.6019/EMPIAR-10028)
  – Cryo-EM structure of the Plasmodium falciparum 80S ribosome ( https://www.ebi.ac.uk/emdb/EMD-2661)
  – Cryo-EM structure of the Plasmodium falciparum 80S ribosome bound to the anti-protozoan drug emetine, large subunit ( https://doi.org/10.2210/pdb3j79/pdb)
  – Cryo-EM structure of the Plasmodium falciparum 80S ribosome bound to the anti-protozoan drug emetine, small subunit ( http://doi.org/10.2210/pdb3j7a/pdb)

## Software availability

Software is available in two different GitHub repositories. We have issued Digital Object Identifier (DOI) for both GitHub repositories.

For the HPC-side code:

- Source code available from: https://github.com/baskerville-hpc/FlowCron-HPC-side
- Archived software available from: DOI ^11^
- License: GPL-3.0

This piece of software should be installed on the HPC in a dedicated project directory, by a Project Investigator or project manager.

For the Globus Flow side code:

- Source code available from: https://github.com/rosalindfranklininstitute/FlowCron-Globus_Flow-side
- Archived software available from: DOI ^10^
- License: Apache-2.0

This piece of software is for the generation of bespoke Globus Flows for a project on a HPC.
