# Supplementary material for: FlowCron - Increasing access to HPC by wrapping Globus into a function-as-a-service
Source: Wellcome Open Res. 2025 Dec 2;10:4. Originally published 2025 Jan 13. [Version 2] doi: 10.12688/wellcomeopenres.23491.2 (PMC11885907; doi:10.12688/wellcomeopenres.23491.2)
Supplement: Peer review history

# Reviewer report: Reviewer response for version 2

**DOI:** 10.21956/wellcomeopenres.28112.r141309

**Published:** 2026-01-05

**Authors:** Weijian Zheng

**Affiliations:** 1 Argonne National Laboratory, Lemont, USA

I think it addressed my suggestions as needed.

Are the conclusions about the tool and its performance adequately supported by the findings presented in the article?

Partly

Is the rationale for developing the new software tool clearly explained?

Yes

Is the description of the software tool technically sound?

Yes

Are sufficient details of the code, methods and analysis (if applicable) provided to allow replication of the software development and its use by others?

Yes

Is sufficient information provided to allow interpretation of the expected output datasets and any results generated using the tool?

Yes

Reviewer Expertise:

High-performance Computing, AI4Science, Machine Learning

I confirm that I have read this submission and believe that I have an appropriate level of expertise to confirm that it is of an acceptable scientific standard.

# Reviewer report: Reviewer response for version 1

**DOI:** 10.21956/wellcomeopenres.25911.r118529

**Published:** 2025-03-06

**Authors:** Christopher Woods

**Affiliations:** 1 University of Bristol, Bristol, England, UK

This is a comprehensive description of the FlowCron tool produced by the authors. The clearly has utility, able to help researchers orchestrate workflows that involve transfer of significant amounts of data. By automating the submission on jobs once the data has been transferred, and automating the copy-back of the data after the job completes, the authors have produced a tool that should improve the efficiency (human time) of running these jobs, and help lower the barrier to use of national digital research infrastructure resources.

 There is an understandable dependency on Globus, and the full utility of the tool really depends on institutions having a Globus subscription. The authors acknowledge this. My hope is that, as other data transfer and federation tools become available, the authors would be willing to explore how their tool could integrate with other systems.

 My only concern with the tool is that the cybersecurity side appears underdeveloped. The tool appears (unless I am mistaken) to submit all jobs under the user account of the PI or project lead who requested that the tool be installed on the DRI. Any jobs submitted under this tool by a third party in a project would bypass necessary user identification and authorization on the DRI and would run under the identity of the PI. Work would be needed to ensure that this could be done in a compliant way (i.e. the PI takes full responsibility for all use of the tool and any cybersecurity breaches), or the authors should explore methods to ensure that federation (e.g. OIDC tokens) are embedded with the job so that the user submitting the job to FlowCron can be independently authenticated and authorized by the DRI. While painful, many DRIs have compliance requirements that users cannot sub-let their accounts or allow others to submit jobs on their behalf. Adding in a federated layer (perhaps based on OIDC tokens) would mitigate this risk and would likely make this tool more interesting for DRI administrators.

Are the conclusions about the tool and its performance adequately supported by the findings presented in the article?

Yes

Is the rationale for developing the new software tool clearly explained?

Yes

Is the description of the software tool technically sound?

Yes

Are sufficient details of the code, methods and analysis (if applicable) provided to allow replication of the software development and its use by others?

Yes

Is sufficient information provided to allow interpretation of the expected output datasets and any results generated using the tool?

Yes

Reviewer Expertise:

Computational biochemistry, computational science, supercomputing, research software engineering, AI

I confirm that I have read this submission and believe that I have an appropriate level of expertise to confirm that it is of an acceptable scientific standard.

# Response

**Published:** 2025-11-28

**Authors:** Dimitrios Bellos

**Affiliations:** Artificial Intelligence and Informatics, The Rosalind Franklin Institute, Didcot, England, UK

Response to **Christopher Woods** (University of Bristol, Bristol, England, UK **):**

**Comment:** *“My only concern with the tool is that the cybersecurity side appears underdeveloped. The tool appears (unless I am mistaken) to submit all jobs under the user account of the PI or project lead who requested that the tool be installed on the DRI. Any jobs submitted under this tool by a third party in a project would bypass necessary user identification and authorization on the DRI and would run under the identity of the PI. Work would be needed to ensure that this could be done in a compliant way (i.e. the PI takes full responsibility for all use of the tool and any cybersecurity breaches), or the authors should explore methods to ensure that federation (e.g. OIDC tokens) are embedded with the job so that the user submitting the job to FlowCron can be independently authenticated and authorized by the DRI. While painful, many DRIs have compliance requirements that users cannot sub-let their accounts or allow others to submit jobs on their behalf. Adding in a federated layer (perhaps based on OIDC tokens) would mitigate this risk and would likely make this tool more interesting for DRI administrators.”*

**Response: **Additional clarification is now provided in the Introduction section. It is now clearly stated that FlowCron’s Globus Flow operates using Globus authentication and thus only users that have access via HPCs’ Globus Collection can use FlowCron’s Globus Flow. Typically, HPCs’ Globus collections can be setup with an OpenID Connect (OIDC) layer, so HPC access policies are followed, thus allowing only authorised users to send data and consequently also use FlowCron. Moreover, access to directories and files via Globus can follow the HPCs’ ACL rules, and thus users that can access an HPC’s Globus collection can only access the appropriate directories/files in the HPC. Indeed, any code executed via a project’s FlowCron is being run using the PI’s/manager’s HPC account; however, only by other project members with access to the same project space in the HPC. It is highly recommended to create a new secondary (service) account with less privileges in the same project and then use this account for the FlowCron installation to comply with the 'principle of least privilege'. This is largely possible due to the way Globus can handle access to an HPC. Lastly, if the HPC’s terms and conditions of use, absolutely prohibit the execution of code from a user via an account that does not belong to the user (PI/manager’s account, project shared account, etc.), then a more sophisticated FaaS solution is required. A good example is the use of Globus Compute. The reasons why we also opted not to use Globus Compute are also stated in the article. All this information is now mentioned in the Introduction section.

# Reviewer report: Reviewer response for version 1

**DOI:** 10.21956/wellcomeopenres.25911.r118528

**Published:** 2025-03-05

**Authors:** Rick Wagner

**Affiliations:** 1 San Diego Supercomputer Center, University of California San Diego, San Diego, USA

This paper describes a tool that packages input data and a Slurm batch script for processing, using a combination of Globus Flows and cron to transfer the data and script to an HPC cluster, execute a processing step via Slurm, and either return the results to the submitting user or track failed jobs. The combination of Globus Flows and the scripts included provide a reliable mechanism for combining data transfer and analysis. The motivation for FlowCron is clearly stated, namely to improve utilization of expensive resources by providing a simplified access mechanism. Unfortunately, there are several issues that prevent me from fully recommending FlowCron and this article, the first being that it does not clearly state near the beginning that it enables a user on an HPC system to allow others to submit batch jobs on their behalf.

 One of the justifications for FlowCron over Globus Compute is the security concern regarding “Remote Code Execution (RCE)”, which is exactly what FlowCron provides through the use of Slurm batch scripts submitted as part of the data transfer. By publishing the resulting Flow to a group, it is enabling submission and execution of arbitrary code through the use of a batch script, and running that code on behalf of the HPC user who deployed FlowCron. This could be used to steal credentials from the user account used to run the batch script. The use of a service account and limiting the data accessible to the individual Units of Work are some of the steps that can reduce the risk of the current design.

 FlowCron is stated to “allows users to analyse their data with minimal training” and it does eliminate the need for users to use SSH to login to the HPC cluster and submit batch scripts directly. However, as mentioned by the user who tested FlowCron, it still requires the user to edit batch scripts with constraints on the use of file paths (see the list under “Operations” on constraints). As described in the section on user testing, having a library of sample batch scripts would be useful for not only FlowCron, but generally for most science use cases. It is not clear that the use and operation of FlowCron is achievable with significantly less training than the typical HPC user experience.

 Architecturally, FlowCron relies on cron, which may not be a reliable service on other HPC systems for users (e.g., the owner of a FlowCron installation). Users will typically run cron on login nodes, which may be reinstalled without preserving a user’s crontab. The Slurm capability scrontab may provide an alternative solution to this issue. Examples from the DKRZ HPC center are provided as references.

 Related to the use of cron, the article states that “FlowCron transfers the responsibilities of installation and upkeep of data processing pipelines from users to HPC systems administrators”, although FlowCron can be installed by HPC system users. This still places the responsibility for maintaining the FlowCron service onto whomever has deployed it.

 Regarding the text and its organization, a diagram like Figure 1 earlier in the article would be helpful to understand what is being described. Also, there are several minor grammatical errors, like missing articles. E.g., “on Globus Website select” should be “on [the] Globus Website select”, and in the next sentence “They can narrow down” the pronoun “they” is unclear. Also, the definition of the term “soft-delete” is repeated. Overall, a large portion of the article reads more like documentation than a scholarly publication.

 In summary, I recommend the following updates:

- Clearly state that FlowCron provides unrestricted access to the HPC account of user who deploys the HPC side to users running the Flow from Globus.
- Provide an estimate of time saved in training and job execution by users of FlowCron compared to typical users, where both have access to similar resources such as a library of sample batch scripts.
- Consider how FlowCron could be deployed on HPC systems that do not allow the use of cron by users.
- Update the text by checking for grammatical errors and duplication, and providing an architecture diagram early in the article.

Are the conclusions about the tool and its performance adequately supported by the findings presented in the article?

Partly

Is the rationale for developing the new software tool clearly explained?

Partly

Is the description of the software tool technically sound?

Partly

Are sufficient details of the code, methods and analysis (if applicable) provided to allow replication of the software development and its use by others?

Yes

Is sufficient information provided to allow interpretation of the expected output datasets and any results generated using the tool?

Yes

Reviewer Expertise:

Astrophysics, turbulence, high-performance computing, cyberinfrastructure, cybersecurity, interactive computing, open-source software governance, Globus, data management, persistent identifiers

I confirm that I have read this submission and believe that I have an appropriate level of expertise to confirm that it is of an acceptable scientific standard, however I have significant reservations, as outlined above.

## References

[1] Reference source

[2] Reference source

# Response

**Published:** 2025-11-28

**Authors:** Dimitrios Bellos

**Affiliations:** Artificial Intelligence and Informatics, The Rosalind Franklin Institute, Didcot, England, UK

Response to **Rick Wagner (**San Diego Supercomputer Center, University of California San Diego, San Diego, USA **):**

**Comment:** *“One of the justifications for FlowCron over Globus Compute is the security concern regarding “Remote Code Execution (RCE)”, which is exactly what FlowCron provides through the use of SLURM batch scripts submitted as part of the data transfer. By publishing the resulting Flow to a group, it is enabling submission and execution of arbitrary code through the use of a batch script, and running that code on behalf of the HPC user who deployed FlowCron. This could be used to steal credentials from the user account used to run the batch script. The use of a service account and limiting the data accessible to the individual Units of Work are some of the steps that can reduce the risk of the current design.”*

**Response:** *“Clearly state that FlowCron provides unrestricted access to the HPC account of user who deploys the HPC side to users running the Flow from Globus.”* Additional clarification is now provided in the Introduction section. It is now clearly stated that FlowCron’s Globus Flow operates using Globus authentication and thus only users that have access via HPCs’ Globus Collection can use FlowCron’s Globus Flow. Typically, HPCs’ Globus collections can be setup with an OpenID Connect (OIDC) layer, so HPC access policies are followed, thus allowing only authorised users to send data and consequently also use FlowCron. Moreover, access to directories and files via Globus can follow the HPCs’ ACL rules, and thus users that can access an HPC’s Globus collection can only access the appropriate directories/files in the HPC. Indeed, any code executed via a project’s FlowCron is being run using the PI’s/manager’s HPC account; however, only by other project members with access to the same project space in the HPC. It is highly recommended to create a new secondary (service) account with less privileges in the same project and then use this account for the FlowCron installation to comply with the 'principle of least privilege'. This is largely possible due to the way Globus can handle access to an HPC. Lastly, if the HPC’s terms and conditions of use, absolutely prohibit the execution of code from a user via an account that does not belong to the user (PI/manager’s account, project shared account, etc.), then a more sophisticated FaaS solution is required. A good example is the use of Globus Compute. The reasons why we also opted not to use Globus Compute are also stated in the article. All this information is now mentioned in the Introduction section.

**Comment:** *“FlowCron is stated to “allows users to analyse their data with minimal training” and it does eliminate the need for users to use SSH to login to the HPC cluster and submit batch scripts directly. However, as mentioned by the user who tested FlowCron, it still requires the user to edit batch scripts with constraints on the use of file paths (see the list under “Operations” on constraints). As described in the section on user testing, having a library of sample batch scripts would be useful for not only FlowCron, but generally for most science use cases. It is not clear that the use and operation of FlowCron is achievable with significantly less training than the typical HPC user experience.”* 

**Response: **The claim for “minimal training” has been changed to “reduced training”. Also, in the Use Cases section it is now clearly stated that the reduction of the users’ training by using a library of template SLURM scripts can be performed independently and thus the positive effect offered by said library cannot be attributed to FlowCron. However, FlowCron reduces the amount of training required to access the HPC via ssh, use Linux terminal commands to navigate to various directories (e.g. personal home directories, project directories, etc.) in the HPC, learn the directory structure of the HPC, monitor the data they have stored in the HPC, safely delete files and clean up their HPC storage. Therefore, the use of FlowCron combined with the use of a library of template scripts can lead to a more user-friendly and self-servicing experience for novice HPC users.

**Comment:** *“Architecturally, FlowCron relies on cron, which may not be a reliable service on other HPC systems for users (e.g., the owner of a FlowCron installation). Users will typically run cron on login nodes, which may be reinstalled without preserving a user’s crontab. The SLURM capability scrontab may provide an alternative solution to this issue. Examples from the DKRZ HPC center are provided as references.”*

**​​​​​​​Response:** *“Consider how FlowCron could be deployed on HPC systems that do not allow the use of cron by users.”* The Introduction section now states the advantages and disadvantages of cron vs scron. Namely, since the frequency of FlowCron’s cron job is high (every few minutes) the use of scron could flood the SLURM queue with jobs run as the user who installed FlowCron. Then this may cause the reduction of the scheduling priority of all HPC jobs submitted by this user, resulting in unnecessary delays. A separate SLURM partition where all login nodes are included, and a separate Quality of Service (QoS) just for SLURM-based cron jobs to avoid the reduction of the scheduling priority of HPC jobs using the normal QoS, may mitigate this issue. However, this requires many changes in the SLURM configuration, causing high administrative costs.  The use of a Unix-based cron by FlowCron relies more on self-service and thus hopefully translates to a set of lower and more accommodating requirements. Nevertheless, the use of Unix-based cron comes with a drawback, namely that FlowCron’s normal operation is dependent on the normal operation of the specific login node used to during its installation. This means that if this specific login node gets deactivated or decommissioned, the cron job needs to be reset on a different login node. The name of the login node used during FlowCron’s installation and the cron command are recorded in log files to help with any troubleshooting. In any case, while this drawback is noteworthy, the probability/frequency of a specific login node getting deactivated or decommissioned is low and in the case of deactivation only temporary, Lastly, FlowCron’s operation can easily be restored even in such cases, by logging to a different login node and executing the cron command thus restarting FlowCron’s cron job.

**Comment:** *“Related to the use of cron, the article states that “FlowCron transfers the responsibilities of installation and upkeep of data processing pipelines from users to HPC systems administrators”, although FlowCron can be installed by HPC system users. This still places the responsibility for maintaining the FlowCron service onto whomever has deployed it.”*

**​​​​​​​Response: **The statement: “FlowCron transfers the responsibilities of installation and upkeep of data processing pipelines from users to HPC systems administrators” in the abstract is now corrected and changed to “FlowCron transfers the responsibilities of installation and upkeep of data processing pipelines from users to HPC project PIs and/or HPC project managers”. With this correction it is clearly stated who is responsible for maintaining the normal operation of FlowCron.

**Comment:** *“Regarding the text and its organization, a diagram like Figure 1 earlier in the article would be helpful to understand what is being described. Also, there are several minor grammatical errors, like missing articles. E.g., “on Globus Website select” should be “on [the] Globus Website select”, and in the next sentence “They can narrow down” the pronoun “they” is unclear. Also, the definition of the term “soft-delete” is repeated. Overall, a large portion of the article reads more like documentation than a scholarly publication.”* *“Update the text by checking for grammatical errors and duplication, and providing an architecture diagram early in the article.”*

**​​​​​​​Response: **Figure 1 is now presented earlier in the document in the Introduction section. Several grammatical errors have been now corrected. The definition of the term “soft-deletion” now only appears once at the first time the term is brought up in the article. Lastly, it is true that the tone of the article differs from a scholarly publication, however this is due to the fact that is not a research article or method article, but a software tool article. Read more Wellcome Open Research article types here: https://wellcomeopenresearch.org/for-authors/article-guidelines/

**Comment:** *“Provide an estimate of time saved in training and job execution by users of FlowCron compared to typical users, where both have access to similar resources such as a library of sample batch scripts.”*

**​​​​​​​Response: **​​​​​​​Unfortunately, we cannot provide exact figures regarding the time reduced due to the automation that FlowCron introduces. FlowCron removes a number of steps that have to be performed manually by the users; however, it is less likely to affect the performance of the code execution and the data transfers via Globus. Since the time required for a human user to execute the various steps manually (initiate Globus transfers of data, submit jobs to the HPC slurm scheduler, etc.) is individual, the amount of time saved may also vary, and no estimate on this would be informative. A survey can be conducted to obtain exact figures; however, we do not have access to a sufficient number of volunteers, and it is not the scope of the present project. Nevertheless, if an organisation is interested in conducting such a survey, we are open to a collaboration. This fact is now clearly stated at the end of the Introduction section.

# Reviewer report: Reviewer response for version 1

**DOI:** 10.21956/wellcomeopenres.25911.r117247

**Published:** 2025-01-29

**Authors:** Weijian Zheng

**Affiliations:** 1 Argonne National Laboratory, Lemont, USA

This report provides a detailed description of how to use Globus to connect data centers and HPC resources, enabling access to high-performance computing in the form of Function-as-a-Service (FaaS). The explanation is clear, well-structured, and should be easy to replicate. The approach appears promising for domain scientists looking to integrate Globus tools into their research workflows.

 I have two suggestions for improvement:

 1. Present performance data, such as function invocation time, to quantify the system’s efficiency.

 2. Reference related works [1-3] that utilize Globus to link facilities, providing additional context and demonstrating broader applicability.

Are the conclusions about the tool and its performance adequately supported by the findings presented in the article?

Partly

Is the rationale for developing the new software tool clearly explained?

Yes

Is the description of the software tool technically sound?

Yes

Are sufficient details of the code, methods and analysis (if applicable) provided to allow replication of the software development and its use by others?

Yes

Is sufficient information provided to allow interpretation of the expected output datasets and any results generated using the tool?

Yes

Reviewer Expertise:

High-performance Computing, AI4Science, Machine Learning

I confirm that I have read this submission and believe that I have an appropriate level of expertise to confirm that it is of an acceptable scientific standard, however I have significant reservations, as outlined above.

## References

[1] Model and Data Management for Machine Learning (M2ML): Integrating Instruments, Edge and HPC for Accelerated Machine Learning.

[2] Bridging data center AI systems with edge computing for actionable information retrieval.

[3] Linking the dynamic picoprobe analytical electronoptical beam line/microscope to supercomputers.

# Response

**Published:** 2025-11-28

**Authors:** Dimitrios Bellos

**Affiliations:** Artificial Intelligence and Informatics, The Rosalind Franklin Institute, Didcot, England, UK

Response to **Weijian Zheng (**Argonne National Laboratory, Lemont, USA **):**

**Comment:** *“Present performance data, such as function invocation time, to quantify the system’s efficiency.”*

**Response: **Unfortunately, we cannot provide exact figures regarding the time reduced due to the automation that FlowCron introduces. FlowCron removes a number of steps that have to be performed manually by the users; however, it is less likely to affect the performance of the code execution and the data transfers via Globus. Since the time required for a human user to execute the various steps manually (initiate Globus transfers of data, submit jobs to the HPC slurm scheduler, etc.) is individual, the amount of time saved may also vary, and no estimate on this would be informative. A survey can be conducted to obtain exact figures; however, we do not have access to a sufficient number of volunteers, and it is not the scope of the present project. Nevertheless, if an organisation is interested in conducting such a survey, we are open to a collaboration. This fact is now clearly stated at the end of the Introduction section.

**Comment:** *“Reference related works [1-3] that utilize Globus to link facilities, providing additional context and demonstrating broader applicability.”*

**Response: **New references (references 10-13) have been included, referencing the work of other researchers that have utilized Globus to link facilities, demonstrating Globus’ broader applicability.
